# Preclinical Evaluation of Synthetic Biology-Driven Engineered *Escherichia coli* Nissle 1917 as a Living Therapeutic for Sustained L‑DOPA Delivery

**DOI:** 10.1021/acssynbio.5c00786

**Published:** 2026-02-02

**Authors:** Ahmed Abdalla, Piyush Padhi, Nicholas Bakes, Ross Thyer, Gary Zenitsky, Huajun Jin, Vellareddy Anantharam, Arthi Kanthasamy, Andrew D. Ellington, Gregory J. Phillips, Anumantha G. Kanthasamy

**Affiliations:** 1 Department of Biomedical Sciences, College of Veterinary Medicine, 1177 Iowa State University, Ames, Iowa 50011-2042, United States; 2 Isakson Center for Neurological Disease Research, Department of Physiology and Pharmacology, 1355 University of Georgia, Athens, Georgia 30602, United States; 3 Department of Veterinary Microbiology & Preventive Medicine, 1177 Iowa State University, Ames, Iowa 50011-2042, United States; 4 Department of Chemical and Biomolecular Engineering, 3990 Rice University, Houston, Texas 77005-1892, United States; 5 Department of Molecular Biosciences, Center for Systems and Synthetic Biology, 12330 University of Texas at Austin, Austin, Texas 78712, United States; 6 Department of Infectious Diseases, College of Veterinary Medicine, 1355 University of Georgia, Athens, Georgia 30602, United States

**Keywords:** L-DOPA, metabolic engineering, live-biotherapeutic, Parkinson’s disease, *Escherichia coli* Nissle 1917, microbiome

## Abstract

Dopamine deficiency resulting from nigrostriatal dopaminergic neuronal damage manifests as extrapyramidal motor symptoms of Parkinson’s disease (PD). Oral tablet dosing of levodopa, administered 3–4 times a day, remains the standard of care due to its tolerability and effectiveness; however, it is prone to deleterious side effects, including off-periods and levodopa-induced dyskinesia after long-term use. Herein, using synthetic biology approaches, we developed and systematically evaluated the feasibility of a probiotic-based live-biotherapeutic system to continuously deliver L-DOPA stably, thereby relieving motor symptoms. Our data demonstrate that our engineered plasmid-based L-DOPA-expressing *Escherichia coli* Nissle 1917 probiotic strain (EcN^2^_LDOPA‑P3_) efficiently produced up to 12,000 ng/mL L-DOPA in vitro. In mouse model systems, EcN^2^_LDOPA‑P3_ readily colonized for up to 48 h, achieved steady-state plasma L-DOPA concentrations, and increased brain L-DOPA and dopamine levels by 1- to 2-fold. Lastly, EcN^2^_LDOPA‑P3_ significantly diminished motor and nonmotor behavioral deficits in a mouse model of PD compared to traditional chemical L-DOPA therapy. These findings support the therapeutic feasibility of a noninvasive, orally administered bioengineered bacterial therapy for the chronic delivery of L-DOPA, which may address limitations associated with current treatment alternatives.

## Graphical abstract

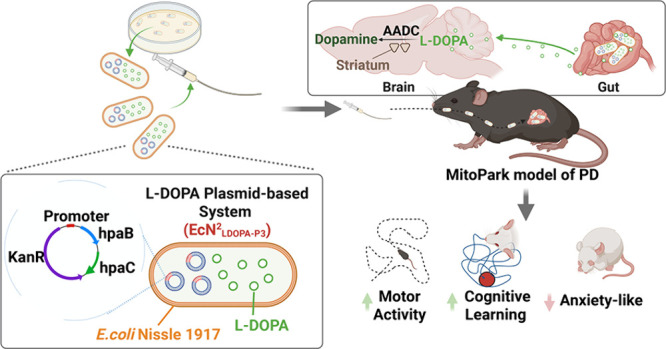

## 1 Introduction

The primary clinical phenotype of PD, which affects 10 million people worldwide,^1^ includes akinesia, rigidity, resting tremor, and postural instability primarily due to the progressive loss of dopaminergic neurons in the substantia nigra (SN) and loss of dopamine (DA) in their striatal axon terminals. Before the onset of motor deficits and decades before a diagnosis of PD, nonmotor symptoms, such as anosmia, autonomic and gastrointestinal (GI) dysfunction, depression, anxiety, and sleep perturbations, equally impair the quality of life and increase healthcare costs.^2–5^ With a global aging population, the incidence of PD is projected to increase, thus potentially imposing enormous societal burdens. Currently, PD remains incurable, and all current treatments target only symptomatic relief.^6,7^

Replenishing DA with L-DOPA has been the gold standard therapy for managing PD motor symptoms since its introduction in the late 1960s.^8,9^ The drug readily enters the blood-brain barrier and dopaminergic neurons, where it is converted to DA by the aromatic L-amino acid decarboxylase (AAADC) enzyme. In the periphery, L-DOPA can also be metabolized by circulating AAADCs and by the host’s gut microbiome.^10^ To limit the proportion metabolized in the periphery to ∼56% and allow more L-DOPA to enter the brain, peripheral AAADC inhibitors (AAADCI), such as carbidopa or benserazide (Bz), are often coadministered. To date, conventional L-DOPA formulations remain the most effective and best-tolerated treatment for relieving PD motor symptoms, as compared to other DA-modulating agents.^11–14^ However, long-term use triggers fluctuating motor responses, the emergence of levodopa-induced dyskinesia (LID), and other nonmotor manifestations. Although the pathophysiology of LID is not entirely understood, the symptoms coincide with the pulsatile stimulation of DA receptors from noncontinuous L-DOPA delivery (3–4 times/day). The ensuing oscillations in plasma L-DOPA concentrations trigger downstream receptor signaling and other neurochemical abnormalities in the basal ganglia.^15–21^

Several strategies have been developed to counter intermittent administration and pulsatile-like stimulation of denervated dopaminergic receptors, such as altering the dosing regimen of L-DOPA formulations, using controlled-release preparations or continuous enteral or subcutaneous infusions, or using monoamine oxidase B (MAO-B) and catechol-*O*-methyl transferase (COMT) inhibitors to extend the half-life of L-DOPA, and using DA agonists.^21,22^ However, higher doses of L-DOPA, dose fractionation, and the use of DA agonists as adjunctive therapy fail to eliminate fluctuations in plasma L-DOPA levels and usually lead to the re-emergence of symptoms,^23,24^ while continuous enteral infusion of L-DOPA remains impractical and cost-prohibitive with high dropout rates.^25,26^ Therefore, developing a more patient-friendly, noninvasive, continuous delivery of L-DOPA in a nonpulsatile manner is needed.

Genetically engineered bacterial therapeutics have emerged as a promising alternative therapy.^27–30^ Several enzymes have been investigated for the commercial biosynthesis of L-DOPA. These include mammalian tyrosine hydroxylases, mushroom tyrosinase, bacterial 4-hydroxyphenylacetate 3-monooxygenase (HpaB), and its flavin adenine dinucleotide (FAD) reductase (HpaC).^31,32^ HpaB is a FAD-dependent monooxygenase that can attack a broad spectrum of compounds with a phenolic group, including l-tyrosine (Figure 1A). In this work, we expressed heterologous *hpaBC* genes in *Escherichia coli* strain Nissle 1917 (EcN), a well-defined probiotic for humans.^33^ Clinical trials using EcN for therapeutic development have been efficacious in patients with chronic idiopathic inflammatory bowel diseases and irritable bowel syndrome.^34,35^ In addition, *hpaBC* expression has been substantially validated in the literature for L-DOPA biosynthesis.^31^ HpaB has a modest cofactor requirement and directly synthesizes L-DOPA from substrate l-tyrosine, which is synthesized de novo through the intact endogenous shikimate–chorismate biosynthetic pathway.^31,36,37^

**Figure 1:**
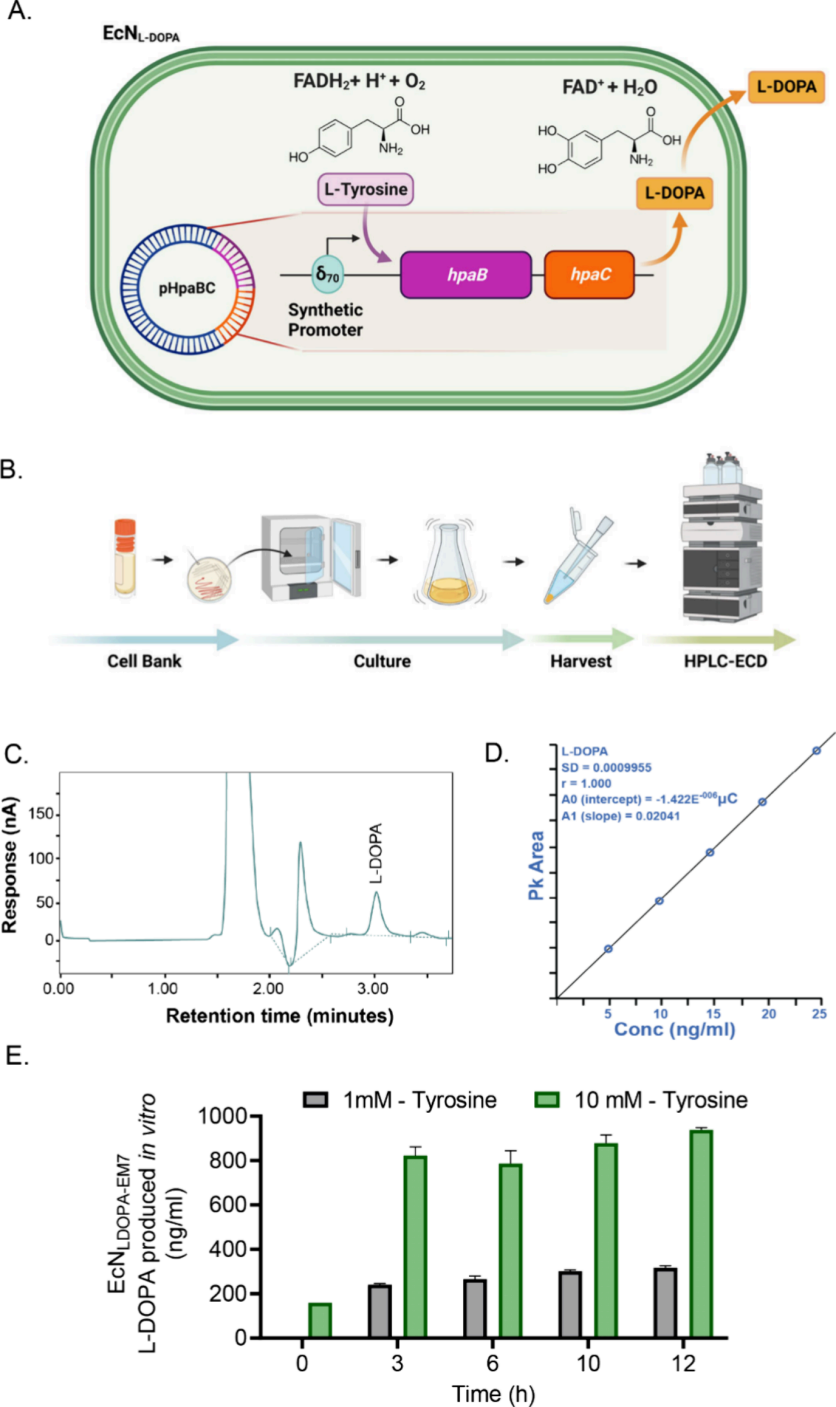
Development of plasmid-based engineered *E. coli* Nissle 1917 for L-DOPA delivery (EcN_LDOPA_). (A) Schematic of engineered EcN strain with L-DOPA plasmid. A recombinant plasmid that expresses the *hpaBC* genes, which encode 4-hydroxyphenylacetate-3-hydroxylase that converts l-tyrosine (Tyr) to L-DOPA, was assembled and used to engineer EcN under control of synthetic δ_70_ promoters. The engineered bacteria use Tyr from surrounding media as well as endogenously synthesized Tyr through the shikimate pathway as a substrate to produce L-DOPA. (B) Schematic of experimental setup for in vitro testing starting with growing EcN_LDOPA_ from frozen stocks to HPLC-ECD testing. (C) HPLC chromatogram profile of bacterial medium. The HPLC peak profile shows a distinct L-DOPA peak identical to the L-DOPA standard curve. (D) L-DOPA standard curve. (E) Mean concentrations of L-DOPA produced in vitro by EcN^1^_L‑DOPA‑EM7_ as detected by HPLC-ECD in the media for two concentrations of Tyr in the media: 1 and 10 mM. Mean concentrations for L-DOPA in the media for 1 mM Tyr shown in gray bars were 0, 240.47, 265.59, 300.85, and 316.56 ng/mL, while concentrations from 10 mM Tyr media (green bars) were 159.4, 822.48, 786.19, 878.99, and 938.47 ng/mL for 0, 3, 6, 10, and 12 h post inoculation, respectively. Data represented as mean ± SEM.

Thus, in this study, we aimed to evaluate the potency of a plasmid-based engineered EcN system expressing hpaBC genes by measuring L-DOPA in vitro and subsequently assessing its therapeutic effects in C57BL/6 (C57) and MitoPark (MP) mouse models of PD, in vivo. For this, we optimized and developed a constitutively expressed, plasmid-based L-DOPA-producing EcN strain (EcN^2^_LDOPA‑P3_) that systematically produces and delivers L-DOPA in a sustained manner, without aberrant fluctuations, while improving both motor and nonmotor symptoms of PD, thus addressing the shortcomings of current DA replacement.

## 2 Results

### 2.1 Construction of a Recombinant EcN Strain (EcN^1^_LDOPA‑EM7_) for the Biosynthesis of L-DOPA In Vitro

In developing cell-based bacterial therapeutics for the continuous microbial production of L-DOPA, we first constructed a plasmid-based EcN strain. The *hpaBC* genes encoding for microbial L-DOPA synthesis were cloned into an RSF1030 expression vector under a synthetic EM7 promoter. Subsequently, the L-DOPA expression plasmid was transformed into EcN and grown on an agar plate with LB-Kan (Figure S1A). We quantitatively assayed L-DOPA production from the first-generation L-DOPA-producing strain (EcN^1^_LDOPA‑EM7_) using HPLC-ECD. Stocks of EcN^1^_LDOPA-EM7_ were grown in LB-Kan media containing l-tyrosine (1 or 10 mM) and ascorbic acid (1 μg/mL) for 12 h at 37 °C. Sterile broth was then collected for HPLC analysis using a C18 column (Figure 1B). The HPLC peak profile of the grown cell-free broth showed a distinct peak at the retention time of 3.01 min (Figure 1C,D), which corresponded to L-DOPA standards, confirming the production of L-DOPA. Over the course of a 12 h experiment, EcN^1^_LDOPA‑EM7_ supplemented with 10 mM l-tyrosine produced significantly higher levels of L-DOPA compared to the media supplemented with 1 mM l-tyrosine (Figure 1E). After 3 h of incubation, EcN^1^_LDOPA‑EM7_ in 10 mM l-tyrosine media reached a L-DOPA mean concentration of 822 ng/mL, while the mean yield achieved in EcN^1^_LDOPA‑EM7_ in 1 mM l-tyrosine was 240 ng/mL. After 12 h incubation, L-DOPA levels reached a mean of 938 ng/mL in 10 mM l-tyrosine media and 316 ng/mL in 1 mM l-tyrosine media (Figure 1E). These results confirmed the capability of an engineered bacterium, EcN^1^_LDOPA‑EM7_, to continuously produce L-DOPA if it was provided with sufficient levels of the substrate l-tyrosine.

### 2.2 EcN^1^_LDOPA‑EM7_ Produced Low Plasma L-DOPA Levels In Vivo without Inducing Toxicity or Significant Gut Microbial Dysbiosis

As a proof of concept, we next tested whether orally administered EcN^1^_LDOPA‑EM7_ can colonize the mouse gut and produce L-DOPA, without inducing toxicity and gut dysbiosis. To assess colonization, we inoculated C57 mice with a single oral dose of 10^9^ CFU EcN^1^_LDOPA‑EM7_ and examined the period live cells were shed (i.e., CFUs), indicating the bacteria’s ability to inhabit the murine gut. Fecal samples were collected daily, weighed, and prepared into pellet suspensions for the spread plate colony count. We detected EcN^1^_LDOPA‑EM7_ in fecal pellets for up to 6 d post-treatment (Figure S1B). Using agarose gel electrophoresis and primers targeted to the unique EcN sequence (Supporting Information Table S1), we confirmed the EcN bacterial strain from selected single colonies (Figure S1C) and in the colon content by qPCR (Figure S1D). Next, to examine whether chronic administration of EcN^1^_LDOPA‑EM7_ increased plasma L-DOPA to human therapeutic levels (300–1600 ng/mL)^38^ or caused any adverse effects in the host tissue, C57 mice were treated with a daily dose of EcN^1^_LDOPA‑EM7_ (10^9^ CFU) or PBS, accompanied by the peripheral decarboxylase inhibitor carbidopa (10 mg/kg, i.p. daily), for 7 d. The animals were also supplemented with 100 mg/kg l-tyrosine daily. After 7 days, the plasma L-DOPA level for EcN^1^_LDOPA‑EM7_-administered animals was notably higher compared to PBS-treated animals (EcN^1^_LDOPA‑EM7:_ 158.2 ng/mL, PBS: 19.26 ng/mL (*p* < 0.02)) (Figure S1E), confirming the in vivo production of L-DOPA. Furthermore, histological, blood chemistry, and fecal taxonomic profiling were conducted to assess toxicity and gut dysbiosis. Blinded histopathological evaluations of the gut segments ileum, cecum, and colon revealed no pathological changes (Supporting Information Table S2 and Figure S1F), suggesting that EcN^1^_LDOPA‑EM7_ treatment did not induce toxicity, inflammation, or neoplastic processes in the mouse gut, while serum chemistry results showed no significant alteration between PBS and EcN^1^_LDOPA‑EM7_-treated mice (Supporting Information Table S3). Moreover, sequence-based 16S taxonomic profiling of mouse fecal samples revealed no significant differences in the key bacterial families, indicating that EcN^1^_LDOPA‑EM7_ did not adversely affect the gut microbiota or cause significant displacement of the established microbiota (Figure S1G). In subsequent experiments, we substituted carbidopa with Bz because of its greater systemic inhibitory potency against peripheral AADC.^39^ Collectively, these results confirm that EcN^1^_LDOPA‑EM7_ can transiently colonize the gut and deliver low levels of L-DOPA in vivo without toxicity or significant gut microbial dysbiosis.

### 2.3 Development of an Improved, Second-Generation L-DOPA Production System

Given that the first-generation EcN^1^_LDOPA‑EM7_ system produced low levels of L-DOPA in vivo and required l-tyrosine supplementation, we next optimized the L-DOPA synthesis by generating an EcN strain that constitutively produced higher levels of L-DOPA without exogenous supplementation. To achieve this, we first designed a synthetic codon-optimized hpaBC operon for *E. coli*, resulting in a 2149-bp sequence containing 437 nucleotide changes, sharing a 79% DNA sequence identity with the original sequence and including 5′ and 3′ sequences to facilitate cloning into the pRham vector (Lucigen). A synthetic gene fragment (IDT) was generated and introduced into the pRham vector under the rhamnose inducible promoter (P_rhaBAD_). Cloning was confirmed by visualizing the oxidative product of L-DOPA, dopachrome under rhamnose, and the absence of color change in the presence of glucose (Figure S2A–C). Next, we replaced the inducible promoter system with constitutive expression-based systems using synthetic constitutive σ^70^ promoters BBa_J23100 (P1), BBa_J23105 (P2), and BBa_J23111 (P3) (Figure 2A and Figure S2D). We quantitatively assayed L-DOPA production by HPLC-ECD from the new transformants (denoted as EcN^2^_LDOPA‑P1_, EcN^2^_LDOPA‑P2_, and EcN^2^_LDOPA‑P3_) over a 12-h growth period from LB culture supernatant (Figure S2E,F) and verified L-DOPA by mass spectrometry analysis (L-DOPA −198.077 *m*/*z*) (Figure 2B). At 12 h, EcN^2^_LDOPA‑P3_ produced 12,000 ng/mL L-DOPA, a substantial yield compared to 2581 ng/mL and 2130 ng/mL observed in EcN^2^_LDOPA‑P1_ and EcN^2^_LDOPA‑P2_, respectively (Figure 2C,D). Moreover, the second-generation EcN^2^_LDOPA‑P3_ strains were capable of producing L-DOPA when grown in LB media lacking l-tyrosine supplementation, compared to wild-type EcN and EcN transformed with an empty vector lacking *hpaBC* (EcN_EV_) (Figure 2E and Figure S2G). This ability is attributable to EcN’s endogenous shikimate–chorismate biosynthetic pathway, which enables de novo l-tyrosine synthesis. As a result, the engineered EcN^2^_LDOPA‑P3_ retains the capacity to produce sufficient intracellular tyrosine, thereby enabling the observed generation of L-DOPA. Collectively, constitutively expressing EcN^2^_LDOPA‑P3_ yielded a 5-fold increase in L-DOPA levels, relative to P1- and P2-engineered EcN strains and did not require additional tyrosine supplementation compared to the EcN^1^_LDOPA‑EM7_ system. Hence, EcN^2^_LDOPA‑P3_ was selected for downstream pharmacokinetic and efficacy studies in rodent models.

**Figure 2:**
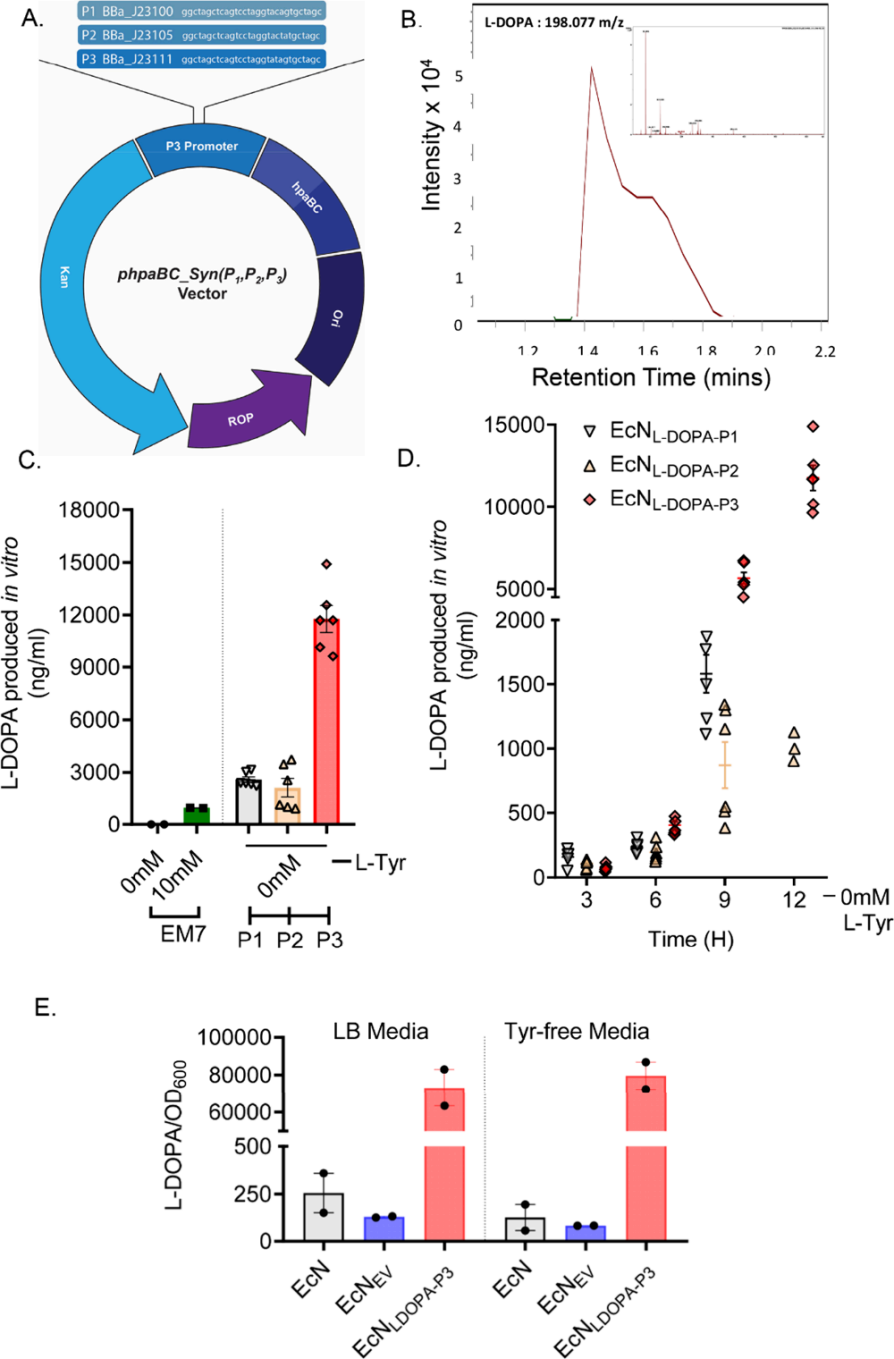
Characterization of engineered plasmid EcN_L‑DOPA_ with constitutive promoters. (A) Plasmid constructs used for expression of *hpaBC* in EcN. Synthetic *hpaBC* genes were expressed from the *pHpaBC_Syn* (P1, P2, and P3)-derivative plasmids as shown. Along with the coding regions for *hpaB* and *hpaC,* additional plasmid features include: Kan, kanamycin resistance; ROP, control of plasmid copy number; Ori, origin of replication. (B) Representative chromatogram of L-DOPA metabolite (*m*/*z* = 198.07) detected by UHPLC-ESI-MS/MS in positive mode multiple reaction monitoring (MRM) mode for analytes mass transitions 152.07 > 139.04 > 107.05 with collision fragmentation voltage set to 20 eV. (C) Quantification of L-DOPA yield. The second-generation L-DOPA-expressing systems (EcN^2^_L‑DOPA‑P1,2,3_) produced significantly higher amounts of L-DOPA compared to the RSF1030-based EcN^1^_L‑DOPA‑EM7_ system after 12-h growth, even without tyrosine (Tyr) supplementation to the LB growth media. ***, *p* < 0.001, by one-way ANOVA, *n* = 4–6. (D) Quantification of L-DOPA from the LB media without additional Tyr (0 mM l-Tyr) from the three EcN strains tested (EcN^2^_L‑DOPA‑P1,2,3_) *n* = 6, measured every 3 h (3, 6, 9, and 12 h). The highest levels of L-DOPA were observed (12,251 ng/mL) in EcN^2^_L‑DOPA‑P3_ after 12-h incubation. (E) Comparison of L-DOPA produced in both regular LB media and l-Tyr-free media in three different EcN strains: wild-type Nissle (EcN), EcN transformed with an empty vector (EcN_EV_), and EcN^2^_L‑DOPA‑P3_, as measured at 12 h and normalized to OD_600_. Presented data from two independent experiments. Data represented as mean ± SEM.

### 2.4 EcN^2^_LDOPA‑P3_ Colonizes the Gut, Produces Therapeutic Levels of L-DOPA, and Increases Brain DA Levels in C57 Mice

To first determine the colonization profile, we inoculated C57 mice with a single dose of EcN^2^_LDOPA‑P3_ in the presence of Bz, collected stool pellets for four consecutive days, and subjected the samples to *hpaBC*-targeted qPCR. We also assessed L-DOPA production, changes in plasma L-DOPA, and striatal L-DOPA/DA post-treatment using HPLC-ECD (Figure 3A–F). After a single oral dose of 10^9^ CFU of EcN^2^_LDOPA‑P3_ or PBS along with the peripheral AADC inhibitor Bz (40 mg/kg, PO every 12 h), significant amounts of EcN^2^_LDOPA‑P3_ were detected in fecal samples for up to 2 d post treatment (4.76 × 10^7^ on day 1 and 1.53 × 10^6^ copy number of *hpaBC* per gram of stool on day 2) (Figure 3B). On day 3, the abundance of EcN^2^_LDOPA‑P3_ in the mouse gut significantly dropped, confirming observations from previous studies^40^ (Figure 3B). EcN^2^_LDOPA‑P3_ was not detected at the baseline or in the PBS vehicle control group. We further quantified EcN^2^_LDOPA‑P3_ from the contents of the duodenum, ileum, cecum, and colon, revealing that EcN^2^_LDOPA‑P3_ could be detected in each of the regions up to 24 h post-treatment, with the highest abundance observed in the cecum within 2 h (Figure 3C).

**Figure 3:**
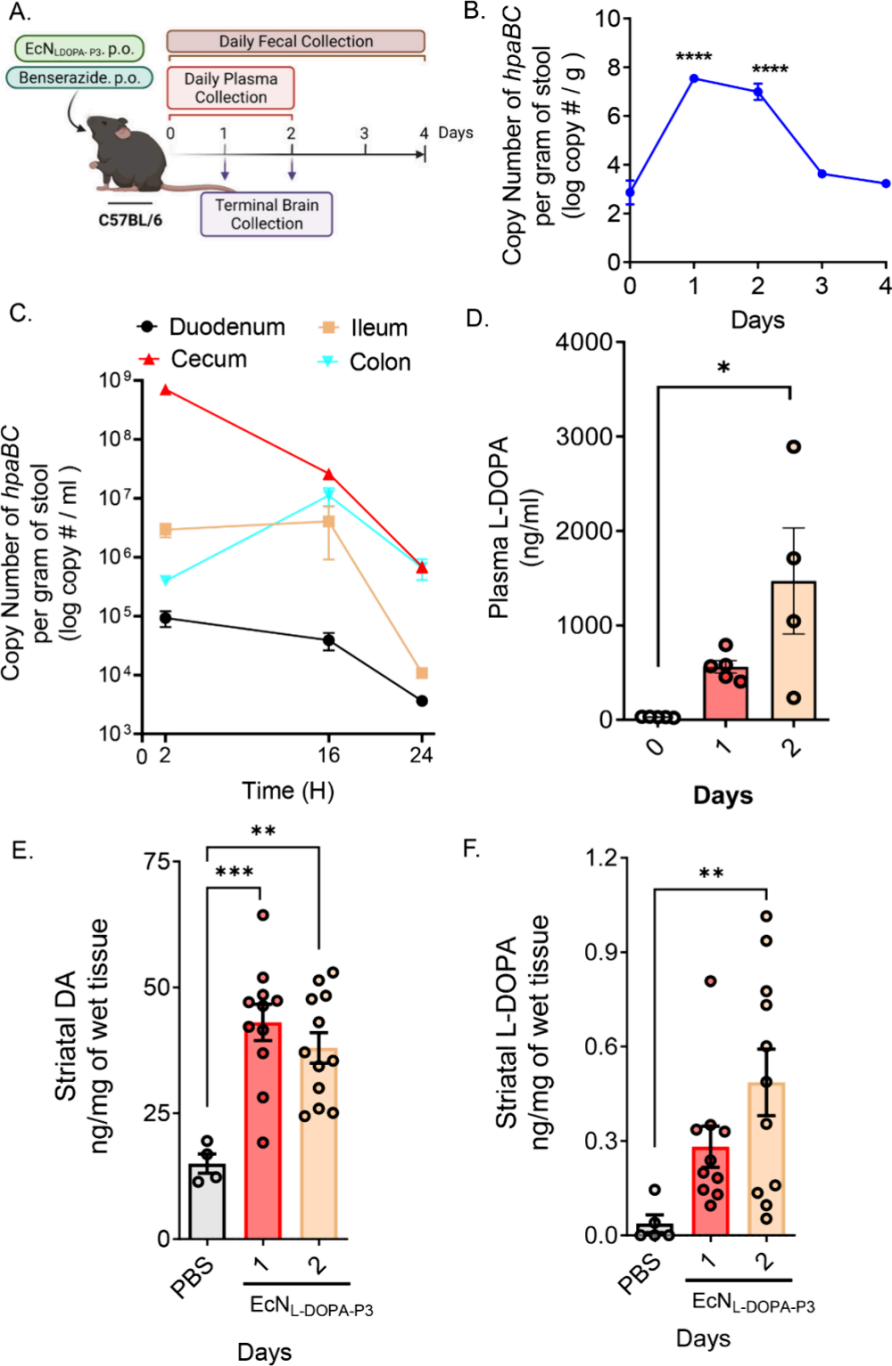
In vivo screening of EcN^2^_LDOPA‑P3_ in healthy C57 mice. (A) Schematic of the experimental design using C57 mice. A single dose of EcN^2^_L‑DOPA‑P3_ (10^9^ CFU) or PBS, in combination with Bz (40 mg/kg PO, every 12 h) administered to mice. (B) Log transform of *hpaBC* DNA copy number per gram feces generating a colonization profile using qPCR (*n* = 6). (C) qPCR analysis of colonization from intestinal content (duodenum, ileum, cecum, and colon) collected at 2, 16, and 24 h post-treatment (*n* = 6 mice, ***, *p* < 0.01 by one-way ANOVA). (D) Plasma L-DOPA concentrations collected from mice (*n* = 4–5) at baseline, 1, and 2 d post EcN^2^_L‑DOPA‑P3_ treatment assayed for L-DOPA production by HPLC-ECD. EcN^2^_L‑DOPA‑P3_ treatment significantly increased plasma levels of L-DOPA over time (28.4 ng/mL at baseline versus 560.6 ng/mL at day 1 and 1470.1 ng/mL at day 2 post-treatment). (E) Mean striatal DA levels in PBS control mice vs mice treated with EcN^2^_L‑DOPA‑P3_ (*n* = 4–6) at 24 and 48 h post-treatment (***p < 0.001). (F) Mean striatal L-DOPA levels analyzed by HPLC-ECD. Data represented as mean ± SEM.

Furthermore, HPLC analysis of plasma L-DOPA and striatal DA levels revealed significant increases in plasma L-DOPA and striatal DA levels at 1 and 2 d post-treatment (Figure 3D–F). EcN^2^_LDOPA‑P3_ treatment significantly increased the mean plasma concentrations of L-DOPA over time from 28.4 ng/mL at baseline to 560.6 ng/mL at 1 d and 1470.1 ng/mL at 2 d post-treatment (Figure 3D), reaching optimal therapeutic plasma levels of L-DOPA relevant in humans.^38^ Concurrently, the mean striatal DA levels increased from 13.56 ng/mg in PBS-treated mice to 20.16 ng/mg at 24 h and 29.67 ng/mg at 48 h after a single dose of EcN^2^_LDOPA‑P3_ (Figure 3E). We also observed an increase in the mean striatal L-DOPA level from 0.03 ng/mg in PBS control mice to 0.28 ng/mg at 24 h and 0.48 ng/mg at 48 h in EcN^2^_LDOPA‑P3_-treated mice (Figure 3F). HPLC analysis of the mouse plasma also revealed a distinct peak of Bz (Supporting Information Figure 3A). Altogether, these results confirm that EcN^2^_LDOPA‑P3_ efficiently colonizes the mouse gut for up to 48 h following treatment, produces therapeutically relevant plasma L-DOPA concentrations, and concurrently elevates striatal L-DOPA and DA levels.

### 2.5 EcN^2^_LDOPA‑P3_ Treatment Produced Similar Plasma Pharmacokinetic Profiles for L-DOPA within the Therapeutic Window in MP and C57 Mice

Next, we systematically compared the pharmacokinetic profiles of EcN^2^_LDOPA‑P3_ with chemically synthesized small-molecule L-DOPA (Chem_L‑DOPA_) in middle-aged MP mice (age 18–20 weeks, representing advanced stages of PD in humans) and age-matched healthy C57 mice. The transgenic MP mouse model exhibits chronically progressive neurodegeneration accompanied by progressive DA loss and motor deficits.^41,42^ Hence, we orally administered a single PO dose of 10^9^ CFU of EcN^2^_LDOPA‑P3_, along with Bz (40 mg/kg, every 12 h), and then compared their plasma concentrations to mice administered a single human-equivalent dose (HED) of Chem_L‑DOPA_ (20.5 mg/kg = 100 mg HED) and Bz (40 mg/kg PO, every 12 h) to both the transgenic MP and healthy C57 mice (Figure 4A). In both mouse strains, EcN^2^_LDOPA‑P3_ rapidly increased plasma L-DOPA concentrations to human therapeutic levels and maintained stable concentrations for up to 30 h in both C57 (Figure 4B) and MP (Figure 4C) mice at varying time points. In contrast, Chem_L‑DOPA_ treatment sharply elevated mean L-DOPA plasma concentrations, exceeding the therapeutic window within the first 4 h. This was followed by a rapid decline to the basal level at 6 h, with concentrations falling below the detection limit at later time points in both C57 and MP mice (Figure 4B,C). Subsequently, sustained plasma L-DOPA in both MP and age-matched healthy C57 mice correlated with the significant increase in striatal DA when compared to PBS control animals (Figure 4C,D). The mean striatal DA for healthy C57 and MP mice increased from 18.3 and 0.66 to 41.19 and 3.3 ng/mL, respectively, 24 h post-EcN^2^_LDOPA‑P3_ administration. These results clearly demonstrate that, unlike traditional administration, EcN^2^_LDOPA‑P3_ treatment can produce stable therapeutic L-DOPA plasma levels up to 30 h post-treatment period and increase striatal DA stores in both C57 and MP mice.

**Figure 4:**
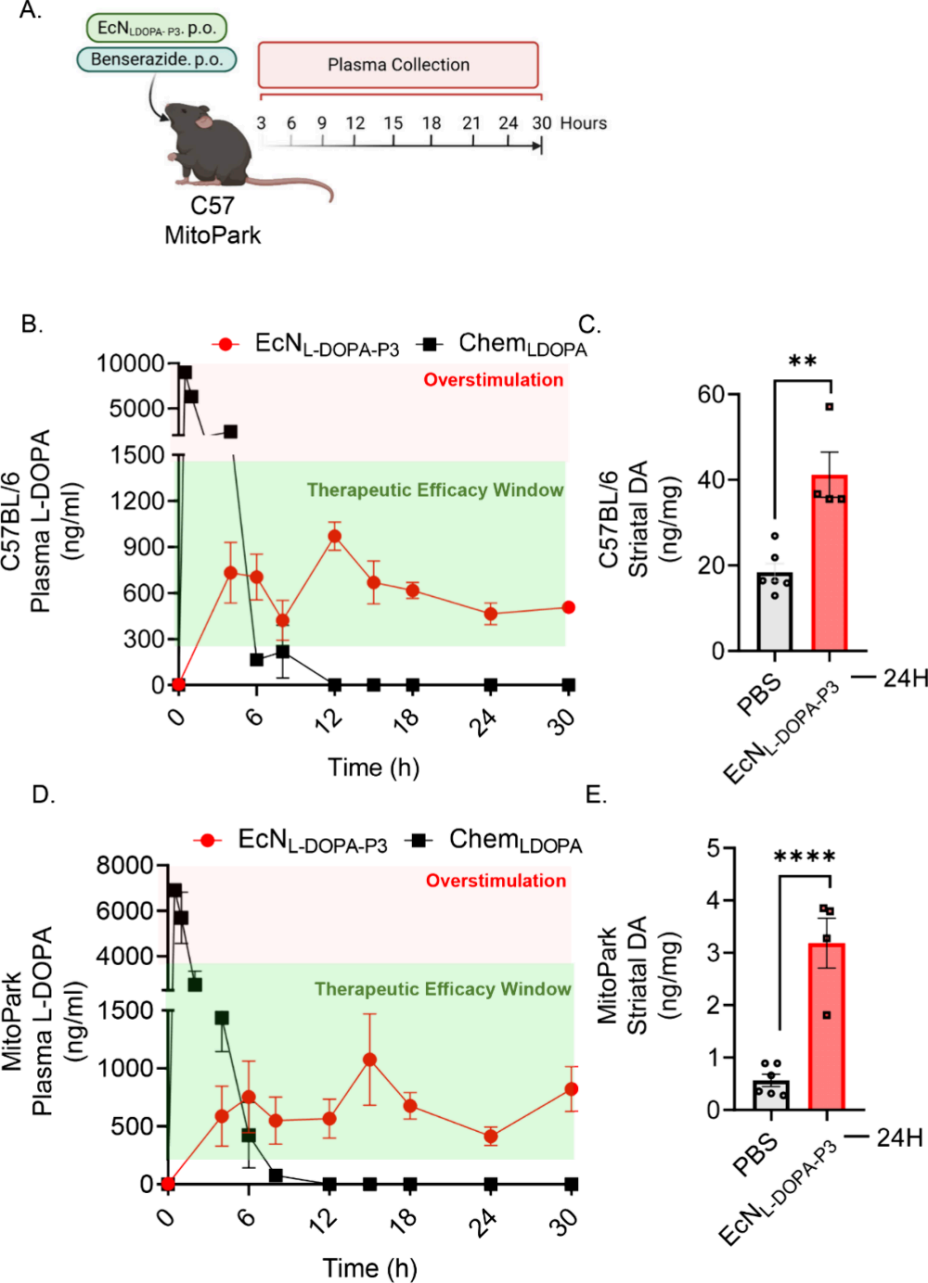
Pharmacokinetic and neurochemical profiling of EcN^2^_L‑DOPA‑P3_ in MitoPark and C57. (A) Schematic of experimental design using both MitoPark and C57 control mice (*n* = 4–6 per time point). (B) Plasma L-DOPA in EcN^2^_LDOPA‑P3_-treated C57 mice shown in red dots remained within the therapeutic efficacy window (green) within 3–30 h, while Chem_L‑DOPA_-treated C57 mice shown in black squares, overstimulated (red) within 0–3 h. (C) Striatal DA level in C57 mice after 24 h of EcN^2^_LDOPA‑P3_ treatment (red bars) compared to PBS control (gray bars). (D) Similarly, plasma L-DOPA in EcN^2^_LDOPA‑P3_-treated MitoPark mice shown in red dots, remained within the therapeutic efficacy window (green) within 3–30 h, while Chem_L‑DOPA_-treated MitoPark mice shown in black squares, overstimulated (red) within 0–3 h. (E) Striatal DA in MitoPark mice after 24 h of EcN^2^_LDOPA‑P3_ treatment (red bars) compared to PBS control (gray bars). Data represented as mean ± SEM.

### 2.6 EcN^2^_LDOPA‑P3_ Treatment Alleviates Neurobehavioral Deficits in MP Mice

We next determined the therapeutic efficacy of oral EcN^2^_LDOPA‑P3_ by assessing locomotor, neuropsychiatric, and cognitive symptoms in the MP PD mouse model. As we previously characterized, MP transgenic mice begin to show significant motor deficits at 10 weeks of age.^43^ Notably, from 12–24 weeks, MP mice show a progressive loss of striatal DA accompanied by pronounced PD-like motor deficits (Figure 5A).^43,44^ Considering its complete clearance from the mouse gut within 48 h (Figure 3B), we administered a dose of either 10^9^ CFU of EcN^2^_LDOPA‑P3_ or PBS in combination with Bz to MP mice (ages 11–16 weeks) every 12 h for 8 weeks. Primarily, behavioral locomotor activity tests confirmed that the increased levels of plasma L-DOPA and striatal DA levels in EcN^2^_LDOPA‑P3_-administered MP mice significantly improved horizontal activity after 1 week of treatment (*p* < 0.0281) (Figure 5B) and sustained the efficacy for over 8 weeks compared to the PBS-treated animals. (Figure 5C). We recently showed that MP mice exhibit progressive deficits in cognitive learning and anxiety-like behaviors.^43^ In the 6-day Morris water maze protocol, EcN^2^_LDOPA‑P3_ improved the cognitive function of MP mice, which exhibited a shorter escape latency compared to PBS control mice on days 2, 3, 4, and 5 (Figure 5D). Similarly, MP mice displayed reduced anxiety-like behavior in the elevated plus maze test, as they spent significantly longer time (Figure 5E) and traveled longer distances (Figure 5F) in the open arms compared to PBS control mice. Moreover, in these animals, EcN^2^_LDOPA‑P3_ increased striatal DA levels from 0.66 to 0.82 ng/mg and replenished its neurochemical stores, as observed from a significant increase in the DA:L-DOPA ratio (Figure 5G,H).

**Figure 5:**
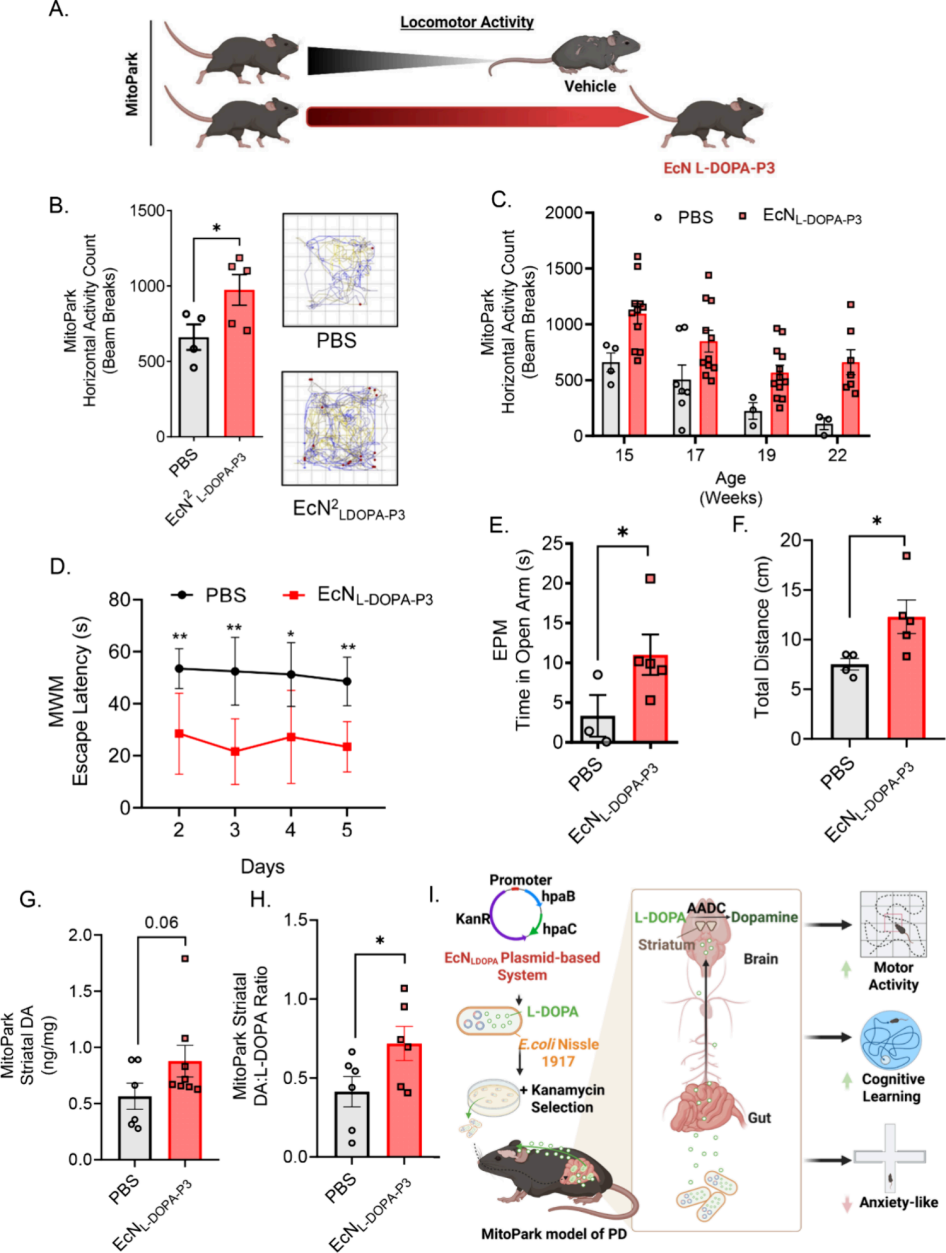
Efficacy of EcN^2^_LDOPA‑P3_ in MitoPark mice. (A) Schematic depiction of progressive nature of MP locomotor deficit. (B) Increased horizontal activity in EcN^2^_LDOPA‑P3_-treated (red bars) mice compared to PBS control group (gray bars) assessed after 1 week of treatment. Representative VersaPlots showing EcN^2^_LDOPA‑P3_ treatment rescued locomotor performance. Lines represent horizontal movement tracks, while red dots represent vertical (rearing) locomotion. (C) Increased horizontal activity in EcN^2^_LDOPA‑P3_-treated group (red bars) compared to PBS control group (gray bars) assessed biweekly. (D) Cognitive dysfunction evaluation in MP mice via MWM test showing improved learning period represented by shortened escape latency in EcN^2^_LDOPA‑P3_-treated group after 8 weeks. Significant decrease in escape latency of MP mice treated with EcN^2^_L‑DOPA‑P3_ (red) compared to PBS-treated group (black). (** *p* < 0.0033, ** *p* < 0.0029, * *p* < 0.0201 and ** *p* < 0.0014). (E) Similarly, reduced anxiety shown in increased time spent in open arms in seconds and total distance for EcN^2^_L‑DOPA‑P3_-treated MP mice (red bars) compared to PBS control (gray bars) (* *p* < 0.0481). (F) Increased DA and (G) DA:L-DOPA ratio after MitoPark was treated with either EcN^2^_LDOPA‑P3_ (red bars) or PBS-treated group (gray bars) demonstrate significant rise in striatal DA stores after EcN^2^_LDOPA‑P3_. Data represented as mean ± SEM.

Collectively, these findings suggest that sustained L-DOPA delivery via EcN^2^_LDOPA‑P3_ enhances dopaminergic neurochemical stores in the brain and provides protection against progressive locomotor, anxiety-like, and cognitive learning deficits in a PD mouse model (Figure 5I).

## 3 Discussion

L-DOPA, in combination with AAADCI, remains the most efficacious dopamine replacement strategy for alleviating motor symptoms in PD patients. However, its therapeutic benefits are significantly limited by the development of motor fluctuations due to unstable plasma levels of the precursor, leading to intermittent nonphysiological stimulation of striatal DA receptors.^16,45–50^ Additionally, repeated daily administration of multiple doses of L-DOPA may further impact a dysregulated dopaminergic system and trigger nonmotor functions that are equally distressing and disabling to patients.^51,52^ To address this challenge, we developed and evaluated the efficacy of a plasmid-based engineered live biotherapeutic designed to deliver L-DOPA in a gradual, consistent, and sustained manner, eliminating the need for frequent dosing. Our results suggest that our engineered plasmid-based biotherapeutic helps avoid fluctuations in plasma L-DOPA concentrations, thereby providing a stable source of L-DOPA for the brain. This steady delivery enables the continuous stimulation of DA receptors, resulting in sustained symptomatic relief without significant side effects. As an added benefit, our novel treatment strategy also alleviates cognitive deficits and anxiety-like behavior in a progressive dopaminergic neurodegeneration model of PD.

To assess the preclinical feasibility of this approach, we constructed multiple EcN variants and tested their ability to produce L-DOPA both in vitro and in vivo. These efforts culminated in the construction of EcN^2^_LDOPA‑P3_. This *E. coli* Nissle strain can produce therapeutically relevant levels of L-DOPA and maintain steady-state plasma concentrations for up to 30 h after a single dose in both healthy and diseased mice. In our in vivo efficacy studies, we used the MP transgenic mouse model of PD to determine its responsiveness to L-DOPA treatment. While classical motor deficits are clearly linked to severe dopaminergic deficits, MP mice also exhibit several nonmotor symptoms of PD, including cognitive deficits and anxiety-like behaviors.^43^ Unstable L-DOPA delivery can impact an already dysregulated system, worsening nonmotor functions. This allows us to explore whether achieving continuous, stable, and nonpulsatile L-DOPA delivery can translate into synchronous improvement of nonmotor symptoms.^51,52^ Indeed, EcN^2^_LDOPA‑P3_ significantly reduced nonmotor symptoms (cognitive deficit and anxiety) of MP mice treated with EcN^2^_LDOPA‑P3_. While additional studies in complementary PD animal models are warranted, our results provide preliminary evidence that EcN^2^_LDOPA‑P3_, as a proof-of-concept engineered gut delivery system, can effectively synthesize and deliver L-DOPA in situ. This approach offers a viable alternative to traditional nonsurgical methods of L-DOPA delivery.^52^

Development of live biotherapeutics is a promising area of investigation for various clinical applications.^53^ Although EcN, the microbial chassis for drug product delivery, has received considerable attention in improving human and animal health,^54,55^ other chassis, such as *Lactococcus lactis*, have been explored.^56^ A significant advantage of the EcN delivery strategy is its transient colonization profile, which enables the monitoring and prediction of both microbial and drug kinetics. Additionally, with a wide variety of synthetic biology tools, EcN is amenable to further genetic manipulation, making it a flexible platform for continual refinement to meet the treatment needs. In EcN^2^_LDOPA‑P3_, *hpaBC* expression is modulated by a constitutive promoter system. By further enhancing expression using different, regulatable, modular, and environment-dependent transcriptional systems, we can adapt a more titratable and fine-tuned approach for human precision-based medicine.

In addition to its use as a drug delivery vehicle, EcN modulates host immune responses,^57^ competitively excluding microbial pathogens,^58^ and improves gut barrier function and mucosal integrity.^54,59–65^ Given that adverse GI symptoms, including motility defects leading to constipation, are manifested by PD patients, EcN has been shown to enhance overall intestinal health and motility^66^ suggesting that our therapy can improve overall intestinal health without causing further dysbiosis, while delivering L-DOPA. Additionally, specific members of the gut microbiota, including *Enterococcus faecalis* and *Eggerthella lenta*, with their endogenous tyrosine decarboxylase (TyrDC), are resistant to the inhibitor Bz.^10,67^ These bacterial enzymes convert L-DOPA to *m*-tyramine, significantly reducing the L-DOPA bioavailability. Conversely, blocking enzyme production increases L-DOPA bioavailability during treatment.^68^ The potential that EcN treatment could minimize these effects should also be considered. For example, EcN can produce antimicrobial compounds (e.g., bacteriocins) that are efficient against *Enterococcus* species, hence may indirectly increase L-DOPA and Bz bioavailability.^69^ While we acknowledge this as a limitation of the current studies, future studies employing targeted metagenomic profiling and metabolite analyses will be needed to elucidate the interactions between EcN^2^_LDOPA‑P3_ and *E. faecalis*. Moreover, since EcN^2^_LDOPA‑P3_ treatment alleviated cognitive deficits and anxiety-like behaviors, additional studies to characterize other nonmotor benefits of EcN_LDOPA_ strategies remain to be explored.

Along with the therapeutic potential, use of EcN^2^_LDOPA‑P3_ as a treatment for PD in humans will require further development to ensure compliance with FDA regulations (docket number: FDA-2010-D-0500). As the current system is a plasmid-based live biotherapeutic, chromosome integration, addition of regulatable transcriptional elements, and inclusion of biocontainment, such as incorporation of “suicide switches” or auxotrophic designs and safety features, for enhancing the clinical safety profile are necessary considerations of human clinical trials.^70^ Nevertheless, this proof of concept provides a feasible strategy for delivering L-DOPA in a sustained manner.

Collectively, the findings from this study provide a promising therapeutic alternative for PD patients that addresses motor fluctuations commonly associated with pulsatile L-DOPA delivery and intermittent DA receptor stimulation^52,71^ of currently prescribed pharmaceutical L-DOPA formulations in both C57 and MP mice. Through rigorous characterization and optimization, the candidate strain described in this manuscript, plasmid-based EcN^2^_LDOPA‑P3_, optimally delivers L-DOPA in a continuous, nonpulsatile manner, resulting in increased striatal DA stores and improvements in motor and nonmotor functions in the MP mouse model of PD. These results provide proof of concept that a bioengineered live therapeutic can effectively regulate brain neurochemical levels, offering a potentially adaptable strategy for treating a range of neurological disorders characterized by neurotransmitter deficiencies.

## 4 Materials and Methods

### 4.1 Chemical Reagents

Lennox L Broth Base (LB Broth catalog no. 12780029) and LB agar powder (Lennox L agar catalog no. 22700025) were purchased from Thermo Fisher Scientific (Carlsbad, CA). Perchloric acid (catalog no. 180-612-186) and sodium metabisulfite (catalog no. S244-3) were purchased from Thermo Fisher Scientific. The PowerUp SYBR Green Master Mix (catalog no. A25742) was purchased from Thermo Fisher Scientific. Glycerol for cell stock preparation (catalog no. G5516), kanamycin sulfate (catalog no. K1377), l-ascorbic acid (catalog no. A4544), ethylenediaminetetraacetic acid (EDTA, catalog no. E6758-100G), L-DOPA (catalog no. D9628), and benserazide hydrochloride (catalog no. B7283) were purchased from Sigma-Aldrich (St. Louis, MO) as were all HPLC standard chemicals including L-DOPA (catalog no. D9628), Bz (catalog no. B7283), dopamine, isoproterenol hydrochloride (catalog no. I6504), and sterile Dulbecco′s phosphate-buffered saline (PBS, catalog no. D8537) for bacterial cell dilution. The DNeasy PowerSoil Pro kit (catalog no. 47014) was obtained from Qiagen (Germantown, MD). Primers and a 2149-bp synthetic gBlocks gene fragment containing the codon-optimized *HpaBC* operon variant were ordered from Integrated DNA Technologies (Coralville, IA).

### 4.2 Bacterial Strain Construction

The EcN strain was kindly provided by Dr. Michael Wannemuehler at Iowa State University. To generate the first-generation biosynthetic plasmid for L-DOPA, the *hpaBC* operon was first amplified from *E. coli* W genomic DNA. This amplicon was ligated into a DNA fragment containing the kanamycin resistance marker and RSF1030 origin of replication derived from plasmid pRSFDuet-1, along with the synthetic constitutive EM7 promoter and the *Vibrio fischeri luxI* terminator using Gibson Assembly.

To reconstruct the L-DOPA-expressing plasmid for improved L-DOPA production, we first cloned a 2149-bp gBlocks gene fragment containing the codon-optimized *hpaBC* operon variant into a commercially available pRham vector (Lucigen, Madison, WI) under control of a rhamnose (Rham) promoter. For this, 100 ng of the *hpaBC* gBlock gene fragment and 25 ng of the linearized pRham vector were added to *E. coli* 10G chemically competent cells and transformed by a heat-shock method. Cells were plated on LB-kanamycin (Kan) (50 μg/mL) and incubated overnight at 37 °C, and transformants were screened on LB-Kan supplemented with 100 μL of 20% L-rhamnose for a dark-pigment-producing phenotype. The presence of dark color in the media is indicative of L-DOPA oxidation to dopachrome with subsequent polymerization to form the pigment melanin.^72^ Correct insertion of the *hpaBC* operon into the pRham vector to form pRham-HpaBC was confirmed by restriction enzyme (*Eco*RI-HF, NEB) digestion of purified plasmid DNA (Monarch Plasmid Miniprep Kit, New England Biolabs) and DNA sequencing. Next, the native Rham promoter of the pRham-HpaBC construct was replaced with multiple constitutive synthetic σ^70^ promoters BBa_J23100, BBa_J23105, and BBa_J23111, which were selected from the iGEM Registry of Standard Biological Parts (http://parts.igem.org/Promoters/Catalog/Constitutive). The three promoters, herein termed P1, P2, and P3, were incorporated into recombinant plasmids to express *hpaBC* by inverse PCR using phosphorylated primers designed to incorporate the 35-bp promoter sequence in place of P_rhaBAD_ on pRham-HpaBC_syn_ (Supporting Information Table 1). Briefly, primer sets P1–P3 were used in separate PCR reactions to amplify a 4.2-kbp DNA fragment using pRham-HpaBC as a template. The amplicons were digested with *Dpn*I and purified by agarose gel elution. One hundred nanograms of purified PCR product was recircularized with T4 ligase, and 2.5 μL of the reaction was chemically transformed into 5-alpha competent cells, with 100 μL of the transformation plated onto LB-Kan and incubated overnight at 37 °C. All plasmid constructs were confirmed by DNA sequencing.

Both the first and second generation of the L-DOPA-producing plasmids were transformed into EcN with the selection on LB-Kan. The EcN_LDOPA_ strain stocks were prepared by mixing 500 μL of overnight cultures with 500 μL glycerol in 2-mL sterile screw top cryotubes and stored at −80 °C.

### 4.3 In Vitro Evaluation of L-DOPA Production

For in vitro evaluation of L-DOPA production, we defrosted glycerol stocks (*n* = 4–6) of the EcN_LDOPA_ strains (EcN^1^_LDOPA‑EM7_ or EcN^2^_LDOPA-(P1, P2, P3)_), streaked them on LB-Kan agar plates, and then incubated them overnight at 37 °C for recovery. Thereafter, we inoculated a single colony in 10 mL of LB medium containing Kan and 1 μg/mL ascorbic acid for 12 h at 37 °C with 230-rpm agitation (Figure 1B). For the EcN^1^_LDOPA‑EM7_ strain, we added l-tyrosine to the medium at 1 and 10 mM and collected samples at 0, 3, 6, and 10 h postinoculation. For EcN^2^_LDOPA‑(P1, P2, P3)_ strains, l-tyrosine was not added to the medium. We further validated the ability of EcN^2^_LDOPA‑P3_ to produce L-DOPA from endogenously synthesized tyrosine by replacing LB media with tyrosine-free media obtained from TEKNOVA Inc., MOPS EZ Rich Defined Medium Kit M2105, minus tyrosine (Hollister, CA). During the growth period, we measured the OD600 of the culture and performed a colony count on an LB-Kan plate to estimate the colony-forming units (CFU) per mL of culture. Samples (500 μL) were collected at each time point by centrifugation at 15,000 rpm for 2 min, and the supernatant was then processed for HPLC analysis of L-DOPA production.

### 4.4 Animals

C57 mice were purchased from Charles River Laboratories. The MitoPark (MP) mouse model was a kind gift of Dr. Nils-Goran Larson at the Max Planck Institute for Biology and Aging in Cologne, Germany, and all MP mice used for this study were bred, maintained, and genotyped at Iowa State University (ISU), Ames, IA, USA. All animals were housed under standard conditions of constant temperature (22 ± 1 °C), humidity (relative 30%), and a 12-h light/dark cycle. Use of the animals and protocol procedures were approved by the Institutional Animal Care and Use Committee (IACUC) at ISU.

### 4.5 Histopathology, Blood Chemistry, and Taxonomic Profiling of C57 Mice

Eight- to 10-week old C57 mice (n = 6, both sexes) were treated with a daily dose of (10^9^ CFU) of EcN^1^_LDOPA‑EM7_ or PBS for 7 days via oral gavage, accompanied by the peripheral decarboxylase inhibitor carbidopa (10 mg/kg, i.p.) daily for 7 d. On top of that, mice were supplemented with an additional l-tyrosine substrate by administering a daily dose of 100 mg/kg l-tyrosine to both treatment groups. Fecal samples were collected once daily for 7 days, weighed, and used for CFU counting on kanamycin agar plates. Part of the daily fecal samples collected were sent for DNA extraction and 16S sequencing analysis. After 7 days, we sacrificed the mice and collected sera, striatal brain regions, and intestinal tissues in 4% paraformaldehyde (PFA) solution. Plasma L-DOPA and brain DA levels were determined by HPLC as previously described.^73^ For histopathology analysis, PFA-fixed mouse gut and organ (ileum, cecum, colon, kidney, liver, and spleen) samples were embedded in paraffin, sectioned, stained with hematoxylin and eosin, and scored for inflammation, edema, stromal collapse, and gland hyperplasia via light microscopy by a board-certified veterinary pathologist in the Veterinary Pathology Department at ISU. The pathologist was blinded to experimental treatments and assigned scores to the sections using the histopathology scoring system for the ileum, colon, and cecum that focuses on several criteria. The scoring is a rising scale from 0 to 5. 0 = the parameter is absent, 1 = parameter is present to a low level (expected for a normal animal), 2 = parameter is mildly increased and is present when multiple high power fields are examined, 3 = parameter is common and is present in most high power fields, 4 = parameter is severe and multiple events are present within the same high power field, and 5 = parameter is severe and so frequent that normal architecture of the tissue is distorted or lost. The scored parameters for the ileum, colon, and cecum were the gland height/villus ratio for cecum and colon (villus/crypt ratio for ileum), which is a ratio of the gland’s length and width. This number is typically between 2 and 6 and varies in different anatomic regions of the colon and cecum. In some inflammatory situations, this number will increase or decrease. Other parameters included assessment or scoring of ulceration, inflammation score and character, stromal collapse, and gland or crypt hyperplasia. Ulceration is a measure of damage to enterocytes lining the glands, crypts, or mucosal surface. Inflammation scores reflect the density of inflammatory cells in the mucosa. Typically, low numbers of inflammatory cells occur in the lamina propria of the ileum, colon, and cecum, where normal tissues usually have a score of 1 (0 is uncommon). In inflammatory situations, this number typically rises. Inflammation character is the type(s) of inflammatory cells in a tissue. In a normal ileum, colon, and cecum, this is typically lymphocytes and plasma cells (mononuclear). In inflammation, these can be lymphocytes, plasma cells, macrophages (mono), neutrophils (PMN), or eosinophils (Eosin). Higher scores indicate erosion and ulceration of the epithelium. Edema indicates tissue fluid expansion of the mucosa and/or submucosa. Stromal collapse suggests a total loss of glands or crypts in a region, with the mesenchymal stroma collapsing on itself. Gland or crypt hyperplasia is an indicator of excessive proliferation within the proliferative compartment of the gland or crypt. In normal tissues, this number is usually 0. In inflammation, this score often increases. Under severe ulceration and stromal collapse, this number may be 0, as glands and crypts are not present. Distribution is a measurement of how frequently lesions are encountered, with 0 indicating no lesions, 1 means a single focus of the section that had the lesion, and 5 means that the lesion was diffuse throughout the section. In the ileum, the villus:crypt ratio replaces the gland height/width ratio. This ratio of villus and crypt height is generally between 1 and 3 and will often decrease during mucosal inflammation.

For blood chemistry analysis, sera were isolated by collecting blood in sterile tubes and allowed to clot for 15 min before centrifugation (2500 rpm) for 10 min. The serum (supernatant) was transferred into a clean sterile tube and submitted for analysis to the Veterinary Pathology Department at Iowa State University for chemistry analysis. Briefly, 100 μL of sample was loaded into the rotor on a VetScan VS2 benchtop clinical analyzer (Abaxis, Union City, CA) for rodent profiling of albumin, alkaline phosphatase (Alk Phos), alanine transaminase (ALT), amylase, total bilirubin, blood urea nitrogen (BUN), calcium, phosphorus, creatinine, glucose, sodium, potassium, and total protein as well as the hemolytic, lipemic, and icteric indices. We evaluated these parameters to determine the extent to which they deviated from the normal range, as an indicator of toxicity.

For taxonomic profiling of the microbiota, genomic DNA was isolated from endpoint fecal samples using four mice per group using a Qiagen DNeasy PowerSoil Pro kit. The purity and concentration of the DNA were measured using a NanoDrop spectrophotometer (Thermo Fisher Scientific, Wilmington, DE) and stored at −80 °C. Isolated DNA was submitted for 16S rRNA gene amplicon sequencing at the Argonne National Laboratory Institute for Genomics and Systems Biology Next Generation Sequencing Core via the V4 region of the bacterial 16S rRNA gene (http://ngs.igsb.anl.gov/). Sequence analysis was done by ISU’s Veterinary Diagnostic Laboratory as previously described^74^ using the fastq.join script and Qiime 1.8. Demultiplexing and quality filtering were then performed using the split_libraries_fastq.py script. The pick_reference_otus_through_otu_table.py script was used for assigning operational taxonomic units (OTU), and taxonomic assignment was performed based on the genes database.^74^

### 4.6 Colonization Studies in C57 Mice

For the initial colonization profiling of the EcN^1^_LDOPA‑EM7_ strain in the C57 mouse gut, we used 15- to 17-week-old mice (n = 4–5) per group for two treatment groups that received either 10^9^ or 10^6^ EcN^1^_LDOPA‑EM7_ cells. Treatment consisted of a single oral dose of 10^9^ or 10^6^ cells per 150 μL mixed with l-tyrosine at 100 mg/mL. Carbidopa at 10 mg/kg was administered i.p. every 12 h. Fecal samples were collected 1, 2, 3, 4, 5, 6, and 7 days post EcN^1^_LDOPA‑EM7_ treatment, and CFU counts on LB-Kan agar plates were recorded daily for 7 d.

For the colonization profiling of the EcN^2^_LDOPA‑P3_ strain, 12- to 16-week-old C57 mice (*n* = 4–6) received a single oral dose (gavage) of 10^9^ CFU of EcN^2^_LDOPA‑P3_ suspended in 150 μL of sterile PBS or 150 μL of PBS alone, along with the peripheral AADCI Bz (40 mg/kg, oral gavage) every 12 h dissolved in 100 μL of water. Fecal samples were collected at 1, 2, 3, and 4 days post-treatment, and EcN^2^_LDOPA‑P3_ copy number was then determined using qPCR with primers targeting *hpaBC* genes.

### 4.7 Pharmacokinetic Profiling of EcN^2^_LDOPA‑P3_ and Chem_L‑DOPA_ in MitoPark and C57 Mice

We evaluated the L-DOPA therapeutic efficacy of EcN^2^_LDOPA‑P3_ treatment in both a PD mouse model and healthy C57 mice. We also quantified plasma L-DOPA and brain DA levels. Middle-aged MP (*n* = 11, 18–20-week-old, representing advanced stages of PD in humans) and age-matched C57 mice (*n* = 12) were randomized by weight into three EcN^2^_LDOPA‑P3_ treatment groups for sacrificing at 21, 24, and 30 h post-treatment, so that eventually, we had *n* = 3–4 per endpoint in our plasma profile. This approach to blood draws was needed to ensure compliance with the IACUC protocols (Figure 4A). Treatment consisted of a single dose of 10^9^ CFU EcN^2^_LDOPA‑P3_ with Bz (40 mg/kg per os (PO) every 12 h). EcN^2^_LDOPA‑P3_ was suspended in 150 μL of sterile PBS and administered via oral gavage (Figure 4A). Within the 30-h study duration, whole blood was collected from the submandibular vein at baseline and at regular intervals (every 3 h) at 3, 6, 9, 12, 15, 18, 21, 24, and 30 h post-treatment. Terminal endpoint blood collection was done via cardiac puncture at 18, 24, and 30 h post-treatment (Figure 4A). Plasma was then isolated by centrifugation immediately after blood collection (2000*g* for 15 min at 4 °C), and the resulting supernatant plasma was collected and stored at −80 °C. Then, plasma L-DOPA levels and brain neurotransmitters were quantified using high-performance liquid chromatography with electrochemical detection (HPLC-ECD).

For the Chem_L‑DOPA_ treatment groups, we similarly used 18–20-week-old MP (*n* = 4) and age-matched C57 mice (*n* = 4). These mice received a single PO dose of Chem_L‑DOPA_ (20.5 mg/kg), suspended in 150 μL of PBS, while Bz (40 mg/kg PO) was given every 12 h dissolved in 100 μL of water, for a total of 30 h.

### 4.8 EcN^2^_LDOPA‑P3_ Efficacy Studies in MitoPark Mice

MP mice (age 11–16 weeks, both sexes, *n* = 4–5) received oral administration of 150 μL of either 10^9^ CFU of EcN^2^_LDOPA‑P3_ or PBS coadministered with Bz (12.5 mg/kg, i.p.) on alternate days for 8 weeks. Animals were assessed for exploratory locomotor activity weekly, 3 to 4 h post-treatment via an open-field test. At the end of the study, mice were sacrificed, and brain neurotransmitters were measured in both PBS and EcN^2^_LDOPA‑P3_ groups as determined in previous publications.^73,75–77^

### 4.9 Locomotor Activity Analysis

For locomotor activity evaluation, we performed an open-field test using the VersaMax system (VersaMax monitor model RXYZCM-16 and analyzer model VMAUSB, AccuScan, Columbus, OH). Using this infrared (IR) tracking system, we recorded spontaneous exploratory activity for a 10-min test period after a 2-min acclimatization period. The system quantifies activity by counting and mapping IR beam breaks within a 20-cm × 20-cm arena.

### 4.10 Morris Water Maze (MWM)

To evaluate the effect of our treatment on cognitive function, we used a 6-d MWM protocol as previously published.^43,77,78^ Briefly, mice were subjected to five 1-min trials daily for the first 5 days. On the first day, we used a visible platform, relocating it between trials to verify that mice can see and swim to it without any motor or visual impairment. On days 2–5, we placed mice in the MWM tank filled with opaque water (prepared by adding white tempera paint) to test their ability to find the hidden platform without changing its location between trials. If the mice failed to locate the hidden platform within 1 min, they were guided to the platform and allowed to remain for 15 s before being removed from the pool. The time required by each mouse to find and mount the platform was measured as escape latency. On day 6, we removed the platform from the tank and subjected mice to a single 1-min probe trial to track their time spent searching in the quadrant that contained the platform during days 2–5. Trials were monitored using ANY-maze tracking software (Stoelting Co., Wood Dale, IL). Intertrial intervals were ≥ 20 min to allow mice to dry out in cages placed on heating pads. Tank water temperature was maintained at 23 ± 1 °C.

### 4.11 Elevated Plus Maze (EPM)

During this test, mice were placed in a plus-shaped apparatus with two open arms and two enclosed arms, and the time spent in the open arms was measured as an inverse correlation of an anxious phenotype.^43,79^ Briefly, mice were placed into the center of the elevated plus maze and video-recorded for 10 min as described.^80^ The video was analyzed by using ANY-maze tracking software.

### 4.12 Preparation of EcN_LDOPA_ Strains for In Vivo Treatment

The EcN_LDOPA_ stocks (EcN^1^_LDOPA‑EM7_ and EcN^2^_LDOPA‑P3_) were thawed and streaked on LB-Kan agar plates. Single colonies were then inoculated into 5 mL of LB-Kan broth with 1 μg/mL ascorbic acid and grown with aeration for 9 h. For the EcN^1^_LDOPA‑EM7_ strain, an additional 10 mM l-tyrosine was also supplemented to the medium. To quantify EcN_LDOPA_ in growth media for in vivo treatment, we used colony counting on LB-Kan agar plates to determine CFU/mL after growth for 9 h before treatment. The cells were then pelleted at 3000*g* for 20 min and then resuspended in 1× PBS according to the desired CFU count per 150 μL. Mice were treated by oral gavage with 150 μL/mouse containing the proposed CFU count of EcN_LDOPA_.

### 4.13 Processing of Fecal and Intestinal Content Samples and qPCR

Fecal samples were collected by transferring mice to cleaned, disinfected plastic cages. Mice were caged individually and allowed to defecate normally before collecting fecal pellets for weighing in sterile 1.5-mL tubes before processing.

After the mice were euthanized, intestinal contents were recovered by dissection of 2–3-cm sections of the duodenum, ileum, cecum, and colon and transferred to sterile plastic plates. Next, 1 mL of sterile PBS was added, and the contents were scraped off the intestinal lumen of each section by using a sterile surgical blade. The contents were recovered in sterile 1.5-mL tubes kept on dry ice before storage at −80 °C until DNA extraction.

Bacterial DNA was isolated from fecal and intestinal content samples as mentioned above (Section 2.5). Isolated DNA underwent qPCR quantification using primer sets targeting *hpaBC* genes (Supporting Information Table 1) (Integrated DNA Technologies, Coralville, IA), which enabled estimation of the EcN_LDOPA_ DNA copy number per gram of feces or gut content. For qPCR, the reaction was prepared using Power SYBR Green reagents (Applied Biosystems) and run in the QuantStudio 3 Real-Time PCR system. For this, we used a synthesized *hpaBC* gene block of known concentration to create an eight-level standard curve run in triplicate. The reaction amplifies DNA (*hpaBC* gene copies) exponentially in the samples. The cycle threshold (Ct) was compared to the standard curve with known DNA concentrations to back-calculate the original copy number of the *hpaBC* gene. All calculations were completed by using the cycle method of quantification in the QuantStudio 3 software. The EcN strain detected in the samples was also verified using primers targeting unique EcN sequences validated in ref 81. For that, qualitative primer sets (Supporting Information Table 1) were used with the SYBR green qPCR reaction as described by Kurtz et al.^81^

### 4.14 HPLC

To quantify the amount of L-DOPA produced by EcN_LDOPA_ in vitro, 500 μL of cultures was collected in sterile 1.5-mL tubes, centrifuged at 15,000 rpm for 1 min at 4 °C, and then transferred immediately to ice. An equal volume of antioxidant solution (perchloric acid HClO_4_ 0.4 M, EDTA 100 mg/mL, sodium metabisulfite Na_2_S_2_O_5_ 50 mg/mL, and HPLC grade water) was added to each sample and stored at −80 °C to help preserve L-DOPA in solution until ready to analyze by HPLC. Plasma L-DOPA and Bz levels were quantified similarly by adding equal volumes of the antioxidant solution containing the internal standard isoproterenol (ISO), a synthetic catecholamine not found in biological tissue.

To conduct assays, samples were thawed on ice and appropriately mixed by brief vortex and transferred into Corning Costar Spin-X centrifuge tube filters (Millipore Sigma, USA catalog #CLS9301) and centrifuged at 14,000 rpm at 4 °C for 15 min. Samples and standards were assayed using the isocratic mobile phase MD-TM (Thermo Fisher Scientific, Waltham, MA, USA) consisting of 80% acetonitrile and 20% water mixed with 0.1% formic acid. Samples were diluted (1:10) into HPLC vials, and an L-DOPA standard curve was generated for 5, 10, 15, 20, and 25 ng/mL concentrations. Plasma samples were run with a standard containing ISO, L-DOPA, Bz, and DA. Samples were loaded to HPLC using a reversed-phase column with a flow rate of 0.6 mL min^–1^ using a Dionex Ultimate 3000 HPLC system (pump ISO-3100SD, Thermo Scientific, Bannockburn, IL) equipped with a refrigerated automatic sampler (model WPS-3000TSL). The electrochemical detection system included a CoulArray model 5600A coupled to an analytical cell (microdialysis cell 5014B) and a guard cell (model 5020). Data acquisition and analysis were performed by using Chromeleon 7 and ESA CoulArray 3.10 HPLC software.

For quantitation of brain DA levels, striatum tissue samples were lysed and analyzed by HPLC after sacrificing the mice following our previously published protocol.^73^ Briefly, the striatal tissue was weighed and lysed by using lysis buffer (0.1 M perchloric acid containing 0.05% Na_2_EDTA and 0.1% Na_2_S_2_O_5_) and ISO as an internal standard. A C-18 reversed-phase column isocratically separated DA, 3,4-dihydroxyphenylacetic acid (DOPAC), and homovanillic acid (HVA).

### 4.15 Liquid Chromatography-Mass Spectrometry (LC-MS/MS)

For sample preparation, 300 μL of EcN_LDOPA_ supernatant media was collected and precipitated by mixing with 2 volumes of methanol, vortexed for 1 min, and followed by 10-min centrifugation at 14,200*g*. After centrifugation, 900 μL of the samples was centrifuged in a 0.22-μM centrifugal filter (Millipore) and stored at −80 °C until 3 μL was injected into the UHPLC-MS/MS.

The chromatographic separation was performed using Phenomenex Luna phenyl-hexyl (100 × 2 mm, 5 μM) with column temperature maintained at 25 °C. The mobile phase consisted of solvent A: 0.1% formic acid, solvent B: 0.1% formic acid in acetonitrile (Fisher-Scientific) with a flow rate of 0.2 mL/min with the following gradient: 0–2 min, 5% B; 2–6 min, 1-segment convex gradient B; 6–8 min, 100% B, 8–10 min, 5% B. Column was equilibrated at 5% B for 5 min before injection. Autosampler temperature was maintained at 4 °C, and MS analysis was conducted on a Bruker Impact II qTOF outfitted with an ESI source in positive polarity. Nitrogen was utilized as the ion source and collision gas. A multiple reaction monitoring mode was initiated to monitor L-DOPA analyte mass transitions (*m*/*z*) 198.07 > 152.07 > 139.04 > 107.05 with collision fragmentation voltage set to 20 eV. All data were analyzed using Compass HyStar (Bruker).

### 4.16 Statistical Analysis

GraphPad 8.0 was used for statistical analysis, with *p* ≤ 0.05 considered statistically significant. One-way ANOVA was used for comparing more than two groups. Two-way ANOVA was used to analyze the occurrence and duration of dyskinesia parameters. In most cases, Tukey’s post analysis was applied. Student’s *t* test was used for comparing two groups.

## Supplementary Material



## Notes

Figure S1: Evaluation of first-generation EcN^1^_LDOPA‑EM7_ in vivo; Figure S2: Development of the second-generation EcN strains for improved L-DOPA production; Figure S3: Benserazide (Bz) administration and dosing validation; Table S1. Oligonucleotide primers used to generate, detect, and quantify *hpaBC* genes and synthetic constitutive promoters; Table S2. Histopathology scores of intestinal sections; Table. S3 Blood chemistry analysis for EcN^1^_LDOPA‑EM7_ -treated C57 mice (PDF)
