# Supplementary material for: Preclinical Evaluation of Synthetic Biology-Driven Engineered Escherichia coli Nissle 1917 as a Living Therapeutic for Sustained L‑DOPA Delivery
Source: ACS Synth Biol. 2026 Feb 2;15(2):748–61. doi: 10.1021/acssynbio.5c00786 (PMC12930508; doi:10.1021/acssynbio.5c00786)
Supplement: Supplementary file 1 [file sb5c00786_si_001.pdf]

# Supplementary Information

## Preclinical Evaluation of Synthetic Biology-Driven Engineered *Escherichia coli* Nissle 1917 as a Living Therapeutic for Sustained L-DOPA Delivery


Ahmed Abdalla[1#], Piyush Padhi[2#], Nicholas Bakes[3], Ross Thyer[4], Gary Zenitsky[2], Huajun Jin[2], Vellareddy Anantharam[2], Arthi Kanthasamy[2], Andrew D. Ellington[5], Gregory J. Phillips[2,6], Anumantha G. Kanthasamy[2*]

[1]Department of Biomedical Sciences, College of Veterinary Medicine, Iowa State University, Ames, IA, USA

[2]Isakson Center for Neurological Disease Research, Department of Physiology and Pharmacology, University of Georgia, Athens, GA, USA

[3]Department of Veterinary Microbiology & Preventive Medicine, Iowa State University, Ames, IA, USA

[4]Department of Chemical and Biomolecular Engineering, Rice University, Houston, TX, USA

[5]Department of Molecular Biosciences, Center for Systems and Synthetic Biology, University of Texas at Austin, Austin, TX, USA

[6]Department of Infectious Diseases, College of Veterinary Medicine, University of Georgia, Athens, GA, USA

[#]These authors contributed equally

*** Correspondence:**
Anumantha Kanthasamy, Ph.D
anumantha.kanthasamy@uga.edu

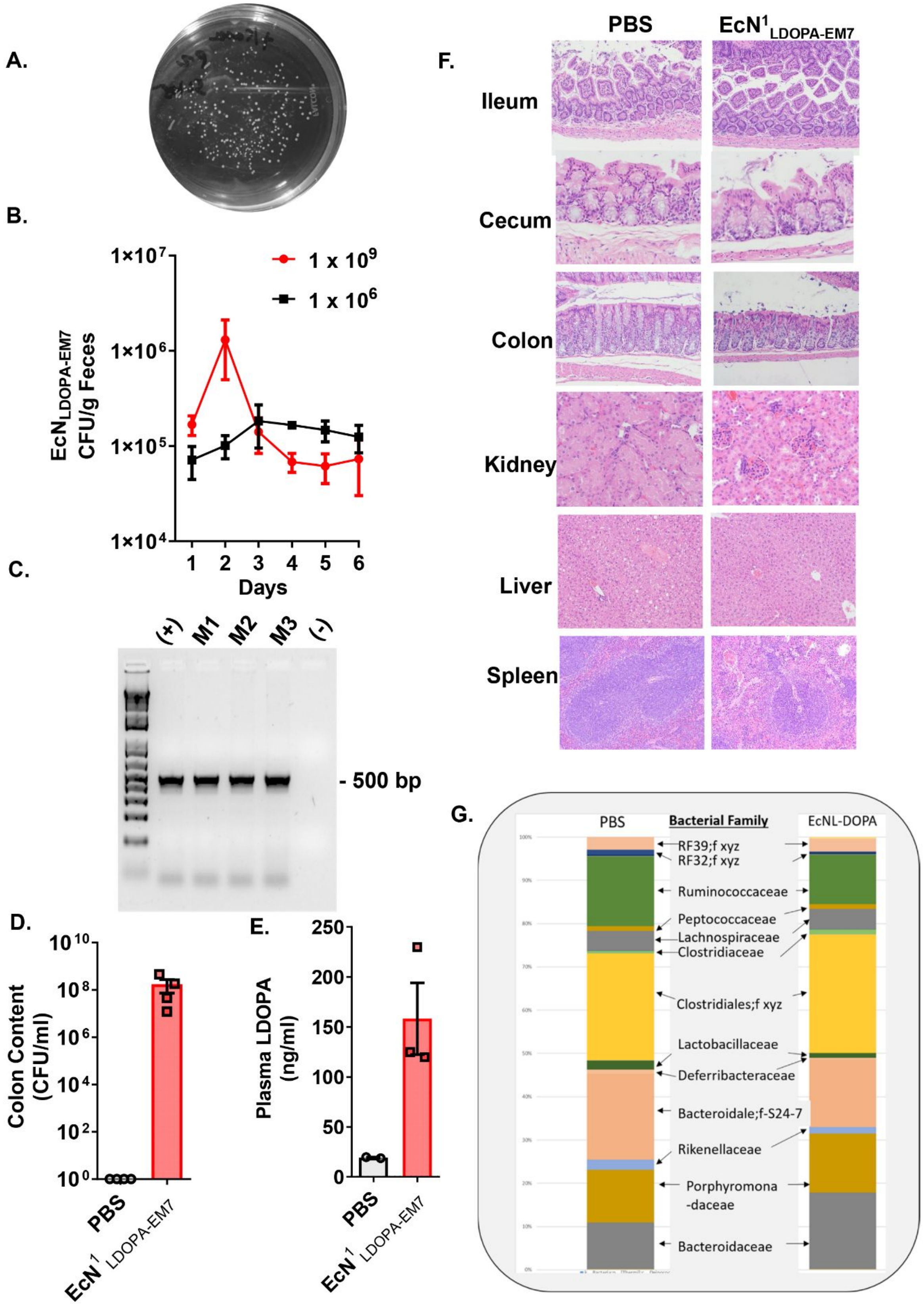
A.
B.
1×10^7
1×10^6
1×10^5
1×10^4
1 x 10^9
1 x 10^6
EcN LDOPA-EM7 CFU/g Feces
1 2 3 4 5 6
Days
C.
(+) M1 M2 M3 (-)
- 500 bp
D.
Colon Content (CFU/ml)
10^10 10^8 10^6 10^4 10^2 10^0
PBS
EcN^1 LDOPA-EM7
E.
Plasma LDOPA (ng/ml)
250 200 150 100 50 0
PBS
EcN^1 LDOPA-EM7
F.
PBS
EcN^1 LDOPA-EM7
Ileum
Cecum
Colon
Kidney
Liver
Spleen
G.
PBS
Bacterial Family
EcNL-DOPA
RF39;f xyz
RF32;f xyz
Ruminococcaceae
Peptococcaceae
Lachnospiraceae
Clostridiaceae
Clostridiales;f xyz
Lactobacillaceae
Deferribacteraceae
Bacteroidale;f-S24-7
Rikenellaceae
Porphyromona -daceae
Bacteroidaceae

**Fig S1: Evaluation of first-generation $EcN^1_{LDOPA\text{-}EM7}$ *in vivo*.** **A)** $EcN^1_{LDOPA\text{-}EM7}$ transformants grown on kanamycin agar plates**.** **B)** Colonization profile generated by colony count in fecal samples after treatment with two different doses of $EcN^1_{LDOPA\text{-}EM7}$ ($10^6$ and $10^9$ CFU). Colonies detected up to 6 d post-treatment are shown, with the highest number at 2 d post-treatment. **C)** L-DOPA genes in plasmid isolated from fecal samples. Amplicons of ~500 bp for a sequence within the *hpaBC* gene are shown. **D)** $EcN^1_{LDOPA\text{-}EM7}$ counts were detected in the colon after 6 d post-treatment while none was detected in the PBS control group. **E)** $EcN^1_{LDOPA\text{-}EM7}$ treatment increased plasma L-DOPA in C57BL/6 mice after daily dosing for 7d compared to the PBS control group ($p<0.02$). **F)** Representative histopathology images for ileum, cecum, and colon besides organs (kidney, liver, and spleen) showing no lesions or inflammatory signs in both of $EcN^1_{LDOPA\text{-}EM7}$-treated and PBS control group. **G)** Taxonomic summary comparison at the family level for PBS control and $EcN^1_{LDOPA\text{-}EM7}$-treated groups. xyz: unnamed family. Genomic DNA isolated from fecal pellets was subjected to PCR amplification of the V4 variable region of 16S rRNA genes and sequenced on the Illumina MiSeq platform. The sequences were analyzed using QIIME (n=4).

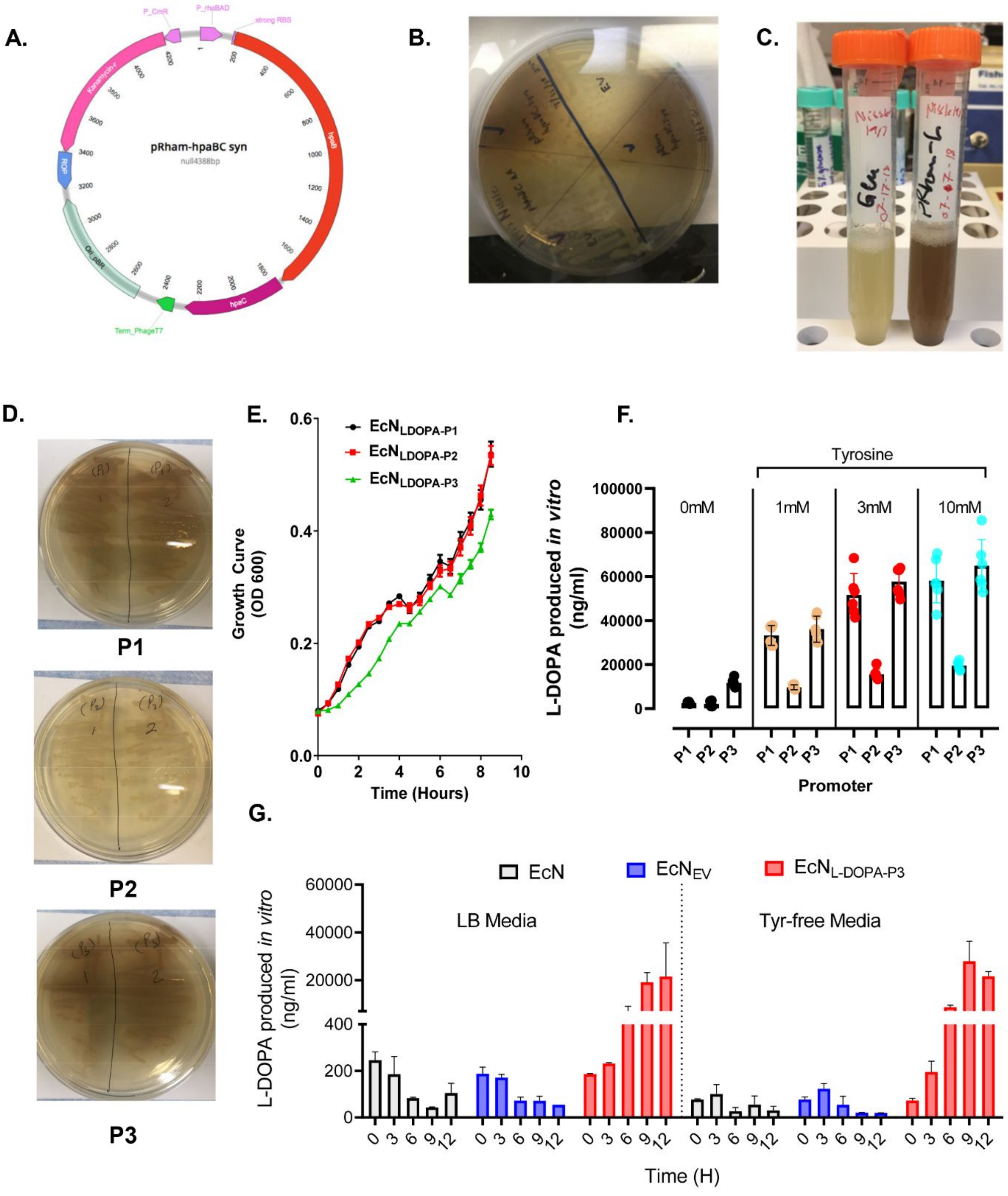
A.
P_CmR
P_rhaBAD
strong RBS
pRham-hpaBC syn
null4388bp
hpaB
hpaC
Term_PhageT7
Ori_pBR
ROP
Kanamycin
B.
C.
D.
P1
P2
P3
E.
EcN$_{LDOPA-P1}$
EcN$_{LDOPA-P2}$
EcN$_{LDOPA-P3}$
Growth Curve (OD 600)
Time (Hours)
F.
Tyrosine
0mM
1mM
3mM
10mM
L-DOPA produced *in vitro* (ng/ml)
Promoter
G.
EcN
EcN$_{EV}$
EcN$_{L-DOPA-P3}$
LB Media
Tyr-free Media
L-DOPA produced *in vitro* (ng/ml)
Time (H)

**Fig S2: Development of the second-generation EcN strains for improved L-DOPA production. A)** pRham plasmid vector construct for inducible *hpaBC* gene expression. **B)** Oxidized L-DOPA produced a distinct dark halo surrounding EcN transformants expressing *hpaBC*, but not the control empty vector. **C)** Induction of *hpaBC* by 0.2% rhamnose (right), while expression is repressed by the addition of 0.15% glucose (left). **D)** Different levels of L-DOPA produced by the 3 promoters P1, 2, and 3. Oxidized L-DOPA production was visualized by dark halos surrounding colonies of the three strains. **E)** $OD_{600}$ (9 h) indicating relatively similar growth properties. **F)** L-DOPA produced in the media after 9-h growth with 4 levels of L-Tyr (0, 1, 3, and 10 mM).**G)** Comparison of L-DOPA produced in both regular LB media and L-Tyr-free media in three different EcN strains: wild-type Nissle (EcN), EcN transformed with an empty vector ($EcN^{2}_{-EV}$), and $EcN^{2}_{L\text{-}DOPA\text{-}P3}$, as measured every 3 h for up to 12 h. Data represented as mean ± SEM.

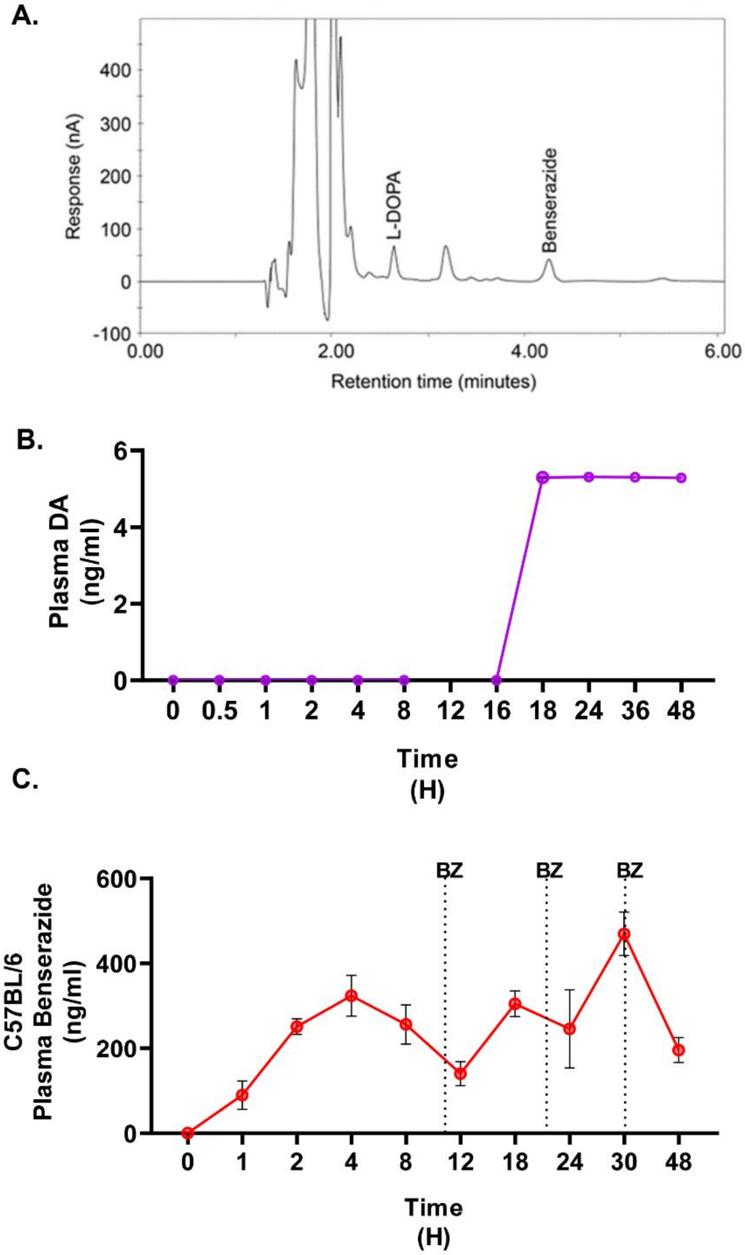


**Fig S3: Benserazide (BZ) administration and dosing validation. A)** Plasma Bz level after a single dose of Bz 12.5 mg/kg i.p. and $10^9$ CFU $EcN^2_{LDOPA\text{-}P3}$ in C57BL/6 mice (n=3-6 per time point). **B)** Mean plasma concentrations of DA (ng/mL) in C57BL/6 mice post single dose of $10^9$ CFU $EcN^2_{LDOPA\text{-}P3}$ and Bz 40 mg/kg PO every 12 hours as quantified by HPLC-ECD. **C)** Mean plasma concentrations of Bz (ng/mL) in C57BL/6 mice as quantified by HPLC-ECD (n=4 at each time-point).

| Name | Target sequence | DNA primer sequence |
|---|---|---|
| P1.S<br>P1.AS | BBa_J23100 (P1)<br>promoter | F- 5’-P -<br>AGGTACTATGCTAGCGAAGGAGATATACATATGAGCAAAGG<br>AGAAG-3’<br>R - 5’-<br>AGGACTGAGCTAGCCGTCAACTGACCATTTAAATCATACCT<br>GACCTCCATAG-3’ |
| P2.S<br>P2.AS | BBa_J23105 (P2)<br>promoter | 5’-P-<br>AGGTACAGTGCTAGCGAAGGAGATATACATATGAGCAAAGG<br>AGAAG-3’<br>5’-<br>AGGACTGAGCTAGCCGTAAACTGACCATTTAAATCATACCT<br>GACCTCCATAG-3’ |
| P3.S<br>P3.AS | BBa_J2311 (P3)<br>promoter | 5’-P-<br>AGGTATAGTGCTAGCGAAGGAGATATACATATGAGCAAAGG<br>AGAAG-3’<br>5’-<br>AGGACTGAGCTAGCCGTCAACTGACCATTTAAATCATACCT<br>GACCTCCATAG-3’ |
| *hpaBC* | *hpaBC* | F - 5’GCAGTCCGTACGATTATCCTTTA3’<br>R- 5'GCGGTCGAAATCACGATAGA3’ |
| N1 (B - 2F, B-2R) | Unique EcN sequence Kurtz et al. (86) | N1 (B-2F: GCAACTGGCCCGTAATTATCC)<br>N1 (B-2R: ACGCATCGCACGTAGGTTT) |

**Table S1. Oligonucleotide primers used to generate, detect and quantify *hpaBC* genes and synthetic constitutive promoters.** Promoter sequences are shown in blue, which are introduced to the plasmids by inverse PCR amplification of pRham-*hpaBC*$_{syn}$, as described in the text.

| Mouse ID | Tissue | Gland Height/Width Ratio | Ulceration | Inflammation Score | Inflammation Character | Edema |
|---|---|---|---|---|---|---|
| 17 | Ileum | 2 | 0 | 1 | Mononuclear | 0 |
| 18 | | 2 | 0 | 1 | Mononuclear | 0 |
| 20 | | 3 | 0 | 2 | Mononuclear | 0 |
| 17 | Cecum | 4 | 0 | 1 | Mononuclear | 0 |
| 18 | | 4 | 0 | 1 | Mononuclear | 0 |
| 20 | | 3 | 0 | 1 | Mononuclear | 0 |
| 17 | Colon | 5 | 0 | 1 | Mononuclear | 1 |
| 18 | | 6 | 0 | 1 | Mononuclear | 0 |
| 20 | | 6 | 0 | 1 | Mononuclear | 0 |

| Mouse ID | Tissue | Stomal Collapse | Crypt Hyperplasia | Distribution |
|---|---|---|---|---|
| 17 | Ileum | 0 | 0 | 0 |
| 18 | | 0 | 0 | 0 |
| 20 | | 0 | 0 | 0 |
| 17 | Cecum | 0 | 0 | 0 |
| 18 | | 0 | 0 | 0 |
| 20 | | 0 | 0 | 0 |
| 17 | Colon | 0 | 0 | 0 |
| 18 | | 0 | 0 | 0 |
| 20 | | 0 | 0 | 0 |

**Table S2. Histopathology scores of intestinal sections.** Inflammatory parameters and their scores for ileum, cecum, and colon are shown.

| Parameter | PBS | $EcN^1_{L\text{-}DOPA\text{-}EM7}$ | Reference Range |
|---|---|---|---|
| Albumin | 4.46 ± 0.18 | 4.22 ± 0.34 | 2.5 – 4.8 |
| Alk Phos | 73.4 ± 12.30 | 62.83 ± 11.27 | 62 - 209 |
| ALT | 36.2 ± 7.26 | 61.17 ± 49.41 | 28 – 132 |
| Amylase | 936 ± 173.48 | 946.67 ± 189.80 | |
| Total Bilirubin | 0.36 ± 0.25 | 0.28 ± 0.04 | 0.1 – 0.9 |
| BUN | 16.6 ± 3.13 | 15.33 ± 2.73 | 18 – 29 |
| Calcium | 11.28 ± 0.23 | 11.47 ± 0.41 | 5.9 – 9.4 |
| Phosphorus | 9.8 ± 0.96 | 10.10 ± 0.79 | 6.1 – 10.1 |
| Creatinine | 0.24 ± 0.09 | 0.22 ± 0.04 | 0.2 – 0.8 |
| Glucose | 274 ± 20.55 | 262.17 ± 34.84 | 90 – 192 |
| Sodium | 15.8 ± 1.64 | 155.67 ± 3.01 | 124 – 174 |
| Potassium | 8.5 ± 0.0 | 8.45 ± 0.12 | 4.6 – 8 |
| Total Protein | 5.82 ± 0.50 | 5.63 ± 0.24 | 3.6 – 6.6 |
| Hemolytic Index | 0 | 0 | |
| Lipemic Index | 0.2 ± 0.45 | 0 | |
| Icteric Index | 0 | 0 | |

**Table S3. Blood chemistry analysis for $EcN^1_{LDOPA\text{-}EM7}$ treated C57 mice.** Blood chemistry parameters with mean values ± SEM for control PBS or $EcN^1_{LDOPA\text{-}EM7}$ treated group (denoted as treatment) are shown. Reference range showing known normal mean value for each parameter is included. Abbreviations: BUN = Blood urea nitrogen; ALK = Alkaline phosphatase; ALT = Alanine transaminase.
